# Exercise-Based Rehabilitation Following Lower-Limb Revascularisation in Patients with Peripheral Arterial Disease: A Scoping Review

**DOI:** 10.3390/diseases14090311

**Published:** 2026-08-26

**Authors:** Shinichi Watanabe, Takayasu Koike, Kenji Tsujimoto, Ryoma Tahara, Tomohiko Kamo, Katsuyoshi Suzuki, Keisuke Suzuki

**Affiliations:** 1 Department of Physical Therapy, Faculty of Rehabilitation, Gifu University of Health Science, 2-92 Higashiuzura, Gifu-City 500-8281, Gifu, Japan; s-watanabe@gifuhoken.ac.jp (S.W.); takayasukoike82@gmail.com (T.K.); tsujiken1260@gmail.com (K.T.); 2 Department of Rehabilitation, National Hospital Organization Nagoya Medical Center, 4-1-1 Sannomaru, Naka-ku, Nagoya 460-0001, Aichi, Japan; 3 Department of Occupational Therapy, Faculty of Rehabilitation, Gifu University of Health Science, 2-92 Higashiuzura, Gifu-City 500-8281, Gifu, Japan; r-tahara@gifuhoken.ac.jp; 4 Department of Physical Therapy, Faculty of Rehabilitation, Gunma Paz University, 1-7-1 Tonyamachi, Takasaki 370-0006, Gunma, Japan; kamo@paz.ac.jp; 5 Department of Rehabilitation, Shizuoka Cancer Center, 1007 Shimonagakubo, Nagaizumi-cho, Sunto-gun 411-8777, Shizuoka, Japan; katsu.suzuki@scchr.jp

**Keywords:** peripheral arterial disease, revascularisation, exercise therapy, rehabilitation, lower-limb ischaemia, chronic limb-threatening ischaemia, walking training

## Abstract

**Objective:** Exercise therapy is recommended for patients with peripheral arterial disease (PAD) to improve walking capacity and functional outcomes. However, rehabilitation programmes after lower-limb revascularisation remain poorly defined. We aimed to map the existing literature on exercise-based rehabilitation following lower-limb revascularisation in patients with PAD and summarise key intervention features and reported outcomes. **Data Sources:** PubMed/MEDLINE, Embase, the Physiotherapy Evidence Database (PEDro), and the Cochrane Central Register of Controlled Trials were systematically searched from inception to identify relevant studies. **Review Methods:** This review followed the Preferred Reporting Items for Systematic Reviews and Meta-Analyses Extension for Scoping Reviews (PRISMA-ScR). Studies involving adult patients with PAD or chronic limb-threatening ischaemia who underwent lower-limb revascularisation and participated in postoperative exercise or rehabilitation programmes were included. Two reviewers independently screened studies and extracted data on study characteristics, intervention components, and outcomes. Findings were synthesised descriptively and presented in tables and narrative summaries. **Results:** Fourteen studies were included. Rehabilitation programmes showed substantial heterogeneity in exercise modality, timing of initiation, intensity, supervision, and duration. Walking-based exercise was the most commonly reported modality. Sessions typically lasted 20–60 min. Exercise frequency ranged from daily inpatient mobilisation or 6 days/week during early rehabilitation to two to five sessions/week in outpatient or home-based programmes, and programme length ranged from inpatient-only rehabilitation to 12 months. The most frequently reported outcomes were measures of walking capacity, including treadmill walking distance and the 6 min walk test. In contrast, limb-related outcomes (e.g., wound healing, limb salvage, and reintervention) and safety outcomes were less frequently reported. **Conclusions:** Exercise-based rehabilitation after lower-limb revascularisation in patients with PAD demonstrates considerable variability in programme characteristics and outcome measures. Although most studies focus on walking performance, key outcomes—particularly limb-related events and safety—remain underreported. Future research should establish standardised postoperative rehabilitation protocols and evaluate a broader range of clinically relevant outcomes, especially in patients with chronic limb-threatening ischaemia.

## 1. Introduction

Peripheral arterial disease (PAD) is a common manifestation of systemic atherosclerosis, affecting over 200 million individuals worldwide and contributing to substantial morbidity, impaired mobility, and increased cardiovascular risk [1,2]. The most advanced form of PAD, chronic limb-threatening ischaemia (CLTI), is characterised by ischaemic rest pain, non-healing ulcers, or gangrene and is associated with a significantly elevated risk of major amputation and mortality [3,4]. Lower-limb revascularisation—including endo-vascular and surgical bypass procedures—remains the cornerstone of treatment, aiming to improve perfusion, relieve ischaemic symptoms, and prevent limb loss in patients with advanced PAD [3,4,5].

Despite successful revascularisation, many patients experience significant postoperative functional limitations. Impaired walking capacity, reduced physical activity, muscle weakness, and delayed mobility recovery are frequently observed in the postoperative period [6,7,8]. Patients with CLTI often present with severe deconditioning, frailty, sarcopenia, and multiple comorbidities, all of which may hinder functional recovery even after successful revascularisation [6,9,10]. Accordingly, improving postoperative functional recovery and mobility has become an increasingly important component of comprehensive PAD management [8,11].

Exercise therapy has been extensively studied in patients with claudication and is strongly recommended in international guidelines due to its ability to improve walking distance, functional performance, and quality of life [4,12,13,14]. Supervised programmes have demonstrated significant improvements in treadmill walking distance and community ambulation in patients with stable PAD [12,13]. However, the applicability of exercise interventions in postoperative settings remains unclear. Patients undergoing revascularisation, particularly those with CLTI, may require careful consideration of rehabilitation timing, wound management, exercise intensity, and safety monitoring [7,15].

Existing studies on postoperative rehabilitation vary widely in exercise modality, programme structure, initiation timing, supervision, and outcome assessment [7,15,16,17]. Restoration of arterial patency does not necessarily translate into functional recovery, and many patients continue to experience limitations despite technically successful revascularisation. From a vascular surgery perspective, clinically relevant outcomes such as graft patency, restenosis, reintervention, and major adverse limb events are critical indicators of procedural success. However, these outcomes have rarely been evaluated in postoperative rehabilitation studies, representing a key gap in the current literature.

Importantly, patients undergoing revascularisation constitute a clinically distinct population, and evidence generated in stable, non-revascularised PAD cannot be directly extrapolated to them. Rehabilitation in the postoperative setting must accommodate surgical wound healing and the integrity of the graft or stent, and the therapeutic objective shifts from promoting collateral adaptation under persistent ischaemia towards restoring function once perfusion has been re-established. Safety monitoring requirements differ accordingly, and patients undergoing revascularisation—particularly those with CLTI—carry a considerably greater burden of frailty, deconditioning, and comorbidity than the ambulatory claudication cohorts in which supervised exercise therapy has been established. These differences in physiology, rehabilitation goals, and safety considerations mean that postoperative rehabilitation warrants investigation in its own right.

We selected a scoping review methodology rather than a systematic review with meta-analysis for three reasons. First, the available evidence base is small and methodologically diverse, encompassing randomised trials, cohort studies, and case series. Second, rehabilitation protocols and outcome measures are highly heterogeneous, so pooling would obscure rather than clarify the differences between interventions. Third, our primary objective is to map what has been done and to identify where evidence is absent, rather than to estimate a pooled treatment effect. A systematic review with meta-analysis would require a degree of standardisation in intervention and outcome reporting that this literature does not currently possess.

Furthermore, previous reviews have primarily focused on patients with claudication rather than postoperative PAD or CLTI populations [17,18,19,20]. Therefore, comprehensive mapping of the available evidence is warranted. We aimed to systematically identify and summarise postoperative exercise-based rehabilitation following lower-limb revascularisation in patients with PAD and characterise the key components of these programmes and their reported outcomes.

## 2. Methods

### 2.1. Study Design

This review was conducted in accordance with the Preferred Reporting Items for Systematic Reviews and Meta-Analyses extension for Scoping Reviews (PRISMA-ScR) guidelines [21]. A scoping review methodology was selected due to the anticipated heterogeneity in study designs, patient populations (including individuals with claudication and CLTI), rehabilitation protocols, and outcome measures. Given this variability and the limited number of studies, a quantitative synthesis or meta-analysis was deemed inappropriate. Instead, this approach allows for comprehensive mapping of the available evidence and identification of key knowledge gaps. The protocol for this scoping review was prospectively registered with the Open Science Framework on 17 December 2025 (https://osf.io/58v7p/; accessed on 26 August 2026).

### 2.2. Participants

Studies involving adult patients (≥18 years) diagnosed with PAD, CLTI, or critical limb ischaemia who underwent lower-limb revascularisation, including endovascular therapy (EVT) or surgical bypass were included. Studies with mixed populations were eligible if adult-specific data could be extracted. The definitions of PAD and CLTI followed widely accepted clinical and guideline-based criteria [3,4]. These populations were included together because they share the defining exposure of this review—lower-limb revascularisation followed by an exercise-based intervention—while differing in prognosis and rehabilitation goals; differences between disease stages were addressed analytically by stratifying studies into claudication-dominant, mixed symptomatic PAD, and CLTI-dominant categories (Supplementary Table S2).

### 2.3. Concept

Studies were included if they described or evaluated exercise-based rehabilitation after lower-limb revascularisation. Eligible studies were required to report at least one quantitative characteristic of the rehabilitation intervention, such as exercise modality (e.g., walking training, cycling, or resistance training), session frequency, session duration, programme length, exercise intensity (e.g., heart rate targets or rating of perceived exertion), mobilisation level, or total exercise dose. Studies describing postoperative care without specific or quantitative information on exercise or rehabilitation were excluded. Interventions were assigned a priori to one or more of four predefined categories: (i) early postoperative mobilisation; (ii) structured exercise programmes without formal supervision; (iii) supervised exercise therapy (SET); and (iv) physical activity promotion or counselling without a prescribed exercise dose. These categories were not treated as interchangeable, as they differ substantially in exercise dose, supervision intensity, and rehabilitation objective; the category assigned to each included study is reported in Table 1.

### 2.4. Context

Studies conducted in diverse clinical settings were considered eligible, including vascular surgery wards, intensive care units, inpatient rehabilitation units, outpatient rehabilitation services, and supervised exercise programmes following revascularisation. Home-based rehabilitation programmes were also eligible if they were explicitly implemented postoperatively. Studies focusing exclusively on preoperative rehabilitation or mixed rehabilitation phases in which postoperative rehabilitation could not be clearly distinguished were excluded.

### 2.5. Types of Sources

This review considered a wide range of evidence sources, including randomised controlled trials (RCTs), quasi-experimental studies, prospective or retrospective cohort studies, case-control studies, and case series with at least 10 patients. Grey literature, including dissertations and health service reports, was also considered [36,37,38]. There were no restrictions regarding publication year or language, provided the full text was accessible. Broad design eligibility is consistent with established scoping review guidance, the purpose of which is to map the extent and nature of the available evidence rather than to appraise a restricted body of comparative studies; restricting inclusion to randomised trials would have excluded most of the postoperative rehabilitation literature and would have concealed the evidentiary gap this review seeks to demonstrate. The threshold of 10 participants was applied pragmatically to exclude single-patient and very small case reports, in which intervention parameters are typically idiosyncratic, while retaining case series large enough to describe a defined protocol; we acknowledge that this threshold is pragmatic rather than derived from a formal methodological standard.

### 2.6. Search Strategy

A comprehensive search strategy was developed to identify both published and unpublished studies. The electronic databases searched were PubMed/MEDLINE (4 February 2026), Embase (4 February 2026), the Physiotherapy Evidence Database (PEDro; 4 February 2026), and the Cochrane Central Register of Controlled Trials (CENTRAL; 4 February 2026). The search combined controlled vocabulary terms and free-text keywords related to PAD, lower-limb revascularisation, and exercise-based rehabilitation [39]. Detailed search strategies for each database are provided in Supplementary Table S1. All database searches were current to 4 February 2026, which is reported here as the uniform final search date. The execution date for each database is given in Supplementary Table S1, and re-running the Embase search on that date returned no additional eligible studies. In addition to database searches, reference lists of the included studies and relevant reviews were manually screened to identify further eligible studies. Grey literature sources, including trial registries and conference abstracts, were also considered to minimise publication bias. Prior to manuscript submission, an updated search was performed to capture studies published after the initial search; no additional eligible studies were identified.

### 2.7. Study Selection

Study selection was conducted using Rayyan, a web-based platform designed to support systematic and scoping reviews. Two reviewers independently screened studies in two stages [40]. First, titles and abstracts were assessed to identify potentially relevant studies. Second, full texts of potentially eligible articles were reviewed to determine final inclusion according to the predefined eligibility criteria. Disagreements between reviewers were resolved through discussion, with a third reviewer consulted if necessary. Rayyan was also used to facilitate systematic review management, identify duplicates, and streamline article screening. The study selection process was documented using a PRISMA-ScR flow diagram, in accordance with recommended reporting standards for scoping reviews [21].

### 2.8. Data Extraction

Data were extracted using Microsoft Excel (Microsoft Corp., Redmond, WA, USA). Two reviewers independently performed data extraction, and any disagreements were resolved through discussion or consultation with a third reviewer. Extracted information included study characteristics (authors, publication year, country, study design, and clinical setting), participant characteristics (sample size, age, sex, and PAD severity), and revascularisation characteristics, including the type of procedure (e.g., endovascular revascularisation, surgical bypass, or hybrid procedures). Details of the exercise interventions were also collected, including exercise modality, timing of initiation after revascularisation, session frequency and duration, total programme length, intensity parameters, and supervision level. Reported outcome measures were extracted, encompassing walking capacity, wound healing, amputation-related outcomes, quality of life, adverse events, rehospitalisation, and mortality.

### 2.9. Data Synthesis

The extracted data were synthesised descriptively to map the characteristics of exercise-based rehabilitation programmes following lower-limb revascularisation. The synthesis focused on identifying patterns in rehabilitation timing, exercise modalities, exercise intensity and dose parameters, supervision level, and reported outcomes [41,42,43,44]. Findings are presented using summary tables and narrative descriptions to provide a comprehensive overview of current evidence and highlight gaps for future research. Additionally, a severity-stratified summary of the included studies was developed to illustrate differences in rehabilitation approaches according to PAD severity. Critical appraisal of individual sources of evidence was not performed, as this review aimed to comprehensively map the existing literature in accordance with the scoping review methodology.

## 3. Results

### 3.1. Study Selection

**Figure 1:**
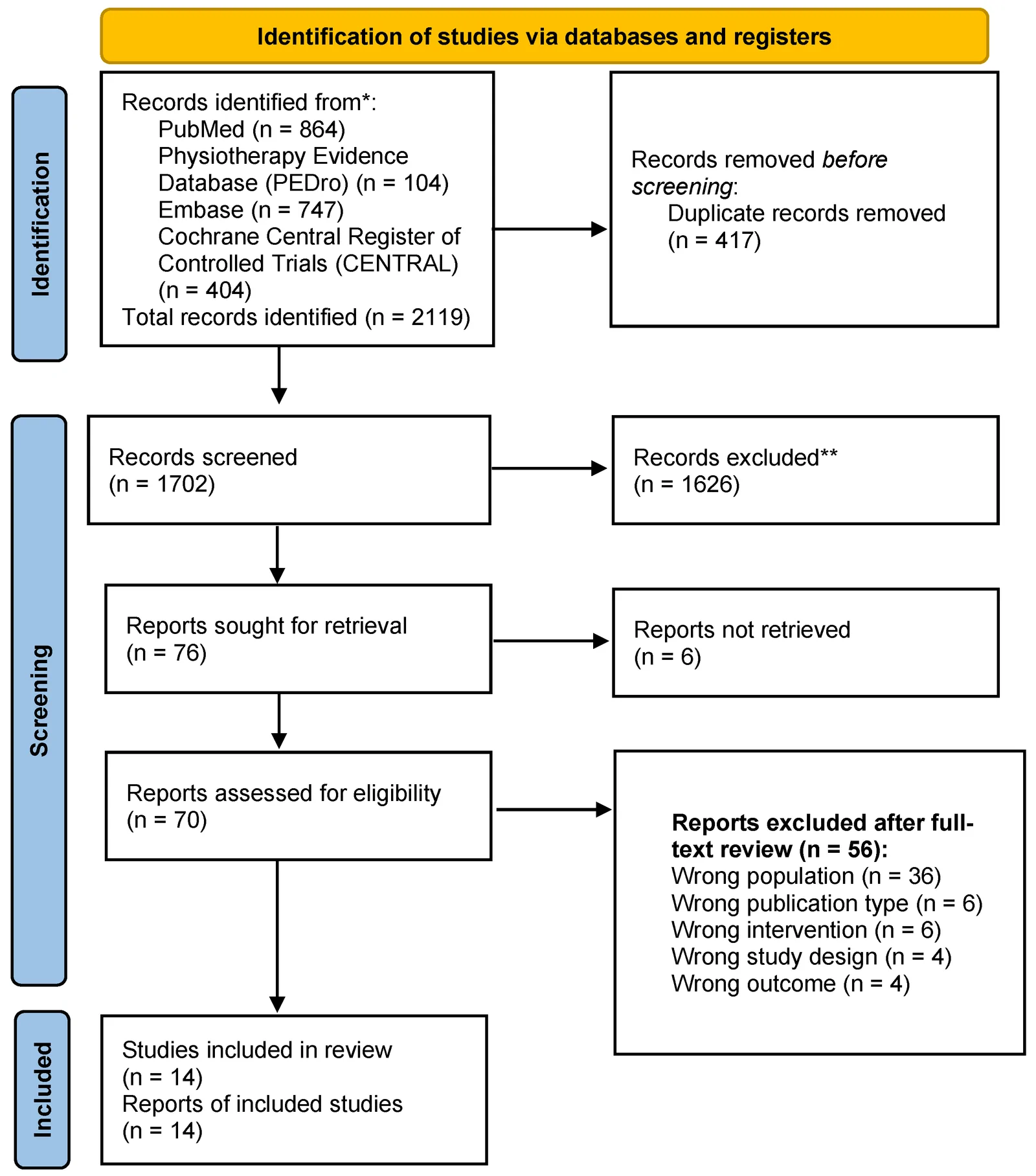
**Study selection process.** Preferred Reporting Items for Systematic Reviews and Meta-Analyses Extension for Scoping Reviews (PRISMA-ScR) flow diagram showing the identification, screening, eligibility assessment, and inclusion of studies. * Consider, if feasible to do so, reporting the number of records identified from each database or register searched (rather than the total number across all databases/registers). ** If automation tools were used, indicate how many records were excluded by a human and how many were excluded by automation tools.

The literature search identified 2119 records across electronic databases (PubMed, *n* = 864; PEDro, *n* = 104; Embase, *n* = 747; CENTRAL, *n* = 404). After removing 417 duplicates, 1702 records remained for title and abstract screening. Following this initial screening, 1626 records were excluded based on predefined eligibility criteria. The great majority of these records were excluded because they concerned populations without PAD, evaluated exercise therapy in patients with PAD who had not undergone revascularisation, described revascularisation without any exercise or rehabilitation component, or were reviews, editorials, or case reports; they were therefore not near-misses for eligibility. A total of 76 full-text reports were sought for retrieval, of which 6 were not obtained. Ultimately, 70 full-text articles were assessed for eligibility. Of these, 56 were excluded for the following reasons: incorrect population (*n* = 36), incorrect publication type (*n* = 6), incorrect intervention (*n* = 6), incorrect study design (*n* = 4), and incorrect outcome (*n* = 4). Fourteen studies met the inclusion criteria and were included in this review. The study selection process is illustrated in Figure 1 (PRISMA flow diagram).

### 3.2. Study Characteristics

The 14 included studies were published between 1989 and 2023 and were conducted across multiple countries in Europe, Asia, and North America (Table 1). The included literature spans more than three decades, and the studies differ systematically by publication era. Studies published before approximately 2010 predominantly evaluated open surgical bypass with inpatient or hospital-based rehabilitation, whereas those published from 2010 onwards predominantly evaluated endovascular revascularisation with outpatient, home-based, or community-based programmes, mirroring the broader shift in PAD management towards endovascular-first strategies [22,23,24,25,26,27,28,29,30,31,32,33,34,35]. Study designs included randomised controlled trials, prospective cohort studies, retrospective cohort studies, and controlled observational studies. Sample sizes ranged from 14 to 218 participants. Most studies enrolled patients with claudication, although several studies also included patients with CLTI. The included studies reported both endovascular interventions and surgical bypass procedures. Earlier studies frequently focused on surgical bypass, whereas more recent studies predominantly examined endovascular therapy. To further illustrate the heterogeneity of the included populations, studies were summarised according to PAD severity categories: claudication-dominant, mixed symptomatic PAD, and CLTI-dominant (Supplementary Table S2).

### 3.3. Characteristics of Exercise-Based Rehabilitation Interventions

**Figure 2:**
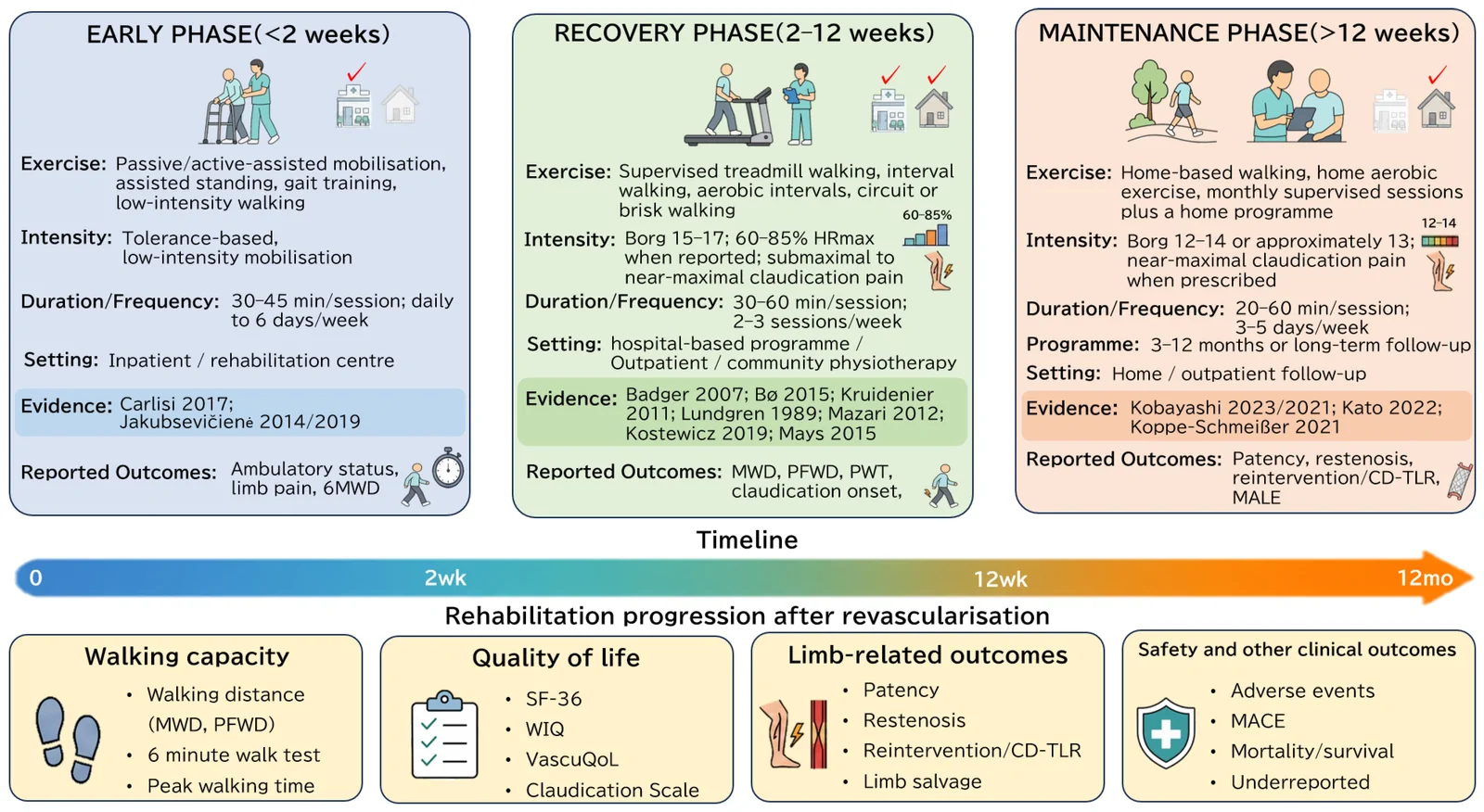
**Rehabilitation progression after lower-limb revascularisation in patients with peripheral arterial disease.** Conceptual framework for postoperative rehabilitation following lower-limb revascularisation, categorised into early (≤2 weeks), recovery (2–12 weeks), and maintenance (>3 months) phases. Rehabilitation typically progresses from low-intensity mobilisation during the early postoperative period to structured supervised exercise during the recovery phase and subsequently to home-based programmes during the maintenance phase. Walking capacity was the most frequently reported outcome, whereas limb-related and safety outcomes were reported less frequently. This framework was derived from patterns identified across the included studies and should not be interpreted as a standardised evidence-based protocol [22,23,24,25,26,27,28,29,30,31,32,33,34,35].

Considerable heterogeneity was observed in exercise-based rehabilitation programmes following revascularisation (Table 2). The most commonly reported modalities were walking-based exercises, including treadmill walking, structured walking programmes, and home-based walking training. Applying the four predefined intervention categories, one study evaluated early postoperative mobilisation [22], four evaluated structured exercise programmes without formal supervision [24,29,30,34], four evaluated supervised exercise therapy alone [25,27,28,33], and five combined an initial supervised phase with a subsequent unsupervised structured programme [23,26,31,32,35]; no study evaluated physical activity promotion or counselling without a prescribed exercise dose (Table 1). Based on reported rehabilitation initiation timing and programme characteristics, interventions can be broadly categorised into three phases (Figure 2). This three-phase framework was derived from patterns identified across the included studies and should be interpreted as a conceptual model rather than a standardised or evidence-based protocol. Differences in rehabilitation approaches according to PAD severity are summarised in Supplementary Table S2. The early phase (0–2 weeks post-revascularisation) typically involves early ambulation and low-intensity mobilisation, including passive mobilisation, assisted standing, and short walking sessions during the immediate postoperative period. The recovery phase (2–12 weeks) generally consists of structured walking programmes, often delivered as supervised exercise therapy (SET) in outpatient or rehabilitation settings. These programmes frequently include treadmill walking, interval walking, and a combination of aerobic and resistance exercises. Exercise intensity is commonly prescribed using Borg scale values, approximately 12–17 across protocols, symptom-limited or submaximal to near-maximal claudication pain, or heart-rate targets corresponding to approximately 60–85% of maximal heart rate (HRmax) when reported. The maintenance phase (>3 months) primarily comprises home-based exercise programmes and long-term physical activity aimed at sustaining functional improvements achieved during earlier rehabilitation phases. Across studies, exercise sessions typically lasted 20–60 min. Frequency ranged from daily inpatient mobilisation or 6 days/week during early rehabilitation to two to five sessions/week in outpatient or home-based programmes, and programme length ranged from inpatient-only rehabilitation to 12 months, with 12-week and 3–6-month programmes commonly reported.

### 3.4. Reported Outcomes

A wide range of outcome measures were reported across the included studies (Table 3). The most frequently reported outcomes were measures of walking capacity, including treadmill walking distance, maximal walking distance, peak walking time, and the 6 min walk test (6MWT). Several studies also evaluated patient-reported outcomes, particularly quality of life, using instruments such as the Short Form-36 (SF-36), the Walking Impairment Questionnaire (WIQ), and the Vascular Quality of Life Questionnaire (VascuQoL). In contrast, limb-related outcomes—including limb salvage, restenosis, and reintervention—were reported less frequently. Similarly, adverse events associated with rehabilitation interventions were rarely documented, with most studies reporting either no major complications or a low incidence of adverse events. Overall, the available evidence suggests that exercise-based rehabilitation following lower-limb revascularisation has primarily focused on improving walking capacity, whereas clinically important outcomes such as limb-related events and safety remain relatively underreported.

Beyond mapping which outcomes were reported, the direction and magnitude of effects were summarised qualitatively across the three principal outcome domains (Table 3). For walking capacity, the most consistently assessed domain, studies reporting walking distance or 6MWT generally favoured the exercise or combined revascularisation-plus-exercise groups, with most studies describing improvements in walking distance, walking time, or 6 min walk performance, although the magnitude of benefit varied across interventions and outcome measures. Patient-reported quality-of-life measures (SF-36, WIQ, VascuQoL) showed broadly concordant improvement, although the magnitude varied and several studies reported non-significant between-group differences. The number of studies formally reporting significant between-group QoL differences was limited; among the eight studies that assessed QoL, approximately half described statistically significant improvements in at least one domain, while the remainder reported non-significant or mixed findings. In contrast, limb-related outcomes (patency, restenosis, reintervention, limb salvage) and safety outcomes were reported in only a minority of studies, and where reported, event rates were low and effect estimates imprecise. Only five of the fourteen included studies reported vascular limb-related outcomes—primarily restenosis, reintervention, or patency—and findings were heterogeneous with no consistent direction of benefit. Adverse events were reported in five studies, generally at low rates, precluding any reliable estimate of the effect of postoperative rehabilitation on safety outcomes. Taken together, the evidence indicates a consistent direction of benefit for walking-based functional outcomes, whereas the effect of postoperative rehabilitation on vascular and safety outcomes cannot be reliably determined from the available data.

## 4. Discussion

PAD is a common manifestation of systemic atherosclerosis and is associated with impaired walking ability, reduced physical activity, and diminished quality of life [1,2]. Patients with PAD often experience progressive functional decline, substantially affecting mobility and independence [6,9,10]. Lower-limb revascularisation procedures, including endovascular therapy and surgical bypass, are widely performed to restore limb perfusion and alleviate ischaemic symptoms, particularly in patients with CLTI [3,4,5]. However, restoration of arterial perfusion does not necessarily translate into optimal functional recovery, and exercise-based rehabilitation has therefore been recognised as a key component of comprehensive PAD management [8,14,20]. From a vascular care perspective, rehabilitation is a critical but often underutilised component, highlighting the need to bridge the gap between procedural success and patient-centred outcomes.

In this scoping review, we mapped the existing literature describing exercise-based rehabilitation following lower-limb revascularisation and identified 14 studies addressing this topic [22,23,24,25,26,27,28,29,30,31,32,33,34,35]. The findings demonstrate considerable variability in rehabilitation programme characteristics, including exercise modality, initiation timing, intensity prescription, supervision, and programme duration. Walking-based exercise was the most frequently reported intervention, consistent with current guideline recommendations emphasising walking training as the cornerstone of PAD rehabilitation [14]. Notably, exercise intensity was prescribed using various approaches, including symptom-limited claudication pain, Borg scale values, or heart rate targets, while programme durations ranged from several weeks to several months. Similar heterogeneity in exercise protocols has been reported in previous systematic reviews of exercise therapy in patients with PAD [16,18]. The severity-stratified summary presented in Supplementary Table S2 further illustrates that rehabilitation strategies differ according to PAD severity. Studies involving claudication primarily focused on improving walking performance and physical activity, whereas studies including patients with CLTI emphasised early mobilisation and basic functional recovery.

Another important observation was the variation in the timing of rehabilitation initiation following revascularisation. Some studies initiated rehabilitation during the early postoperative period, whereas others implemented structured exercise programmes several weeks after revascularisation in outpatient settings. These differences may reflect variations in postoperative care pathways, concerns regarding wound healing, and differences in patient populations, particularly between those with claudication and those with CLTI [3,5]. Early mobilisation strategies have been proposed to prevent postoperative deconditioning and improve functional recovery in vascular surgery populations [7,17]. However, the optimal timing and progression of rehabilitation after revascularisation remain unclear and require further investigation.

This review also demonstrated that walking capacity was the most frequently reported outcome measure. Improvements in walking performance, including treadmill walking distance and the 6MWT, are widely recognised therapeutic targets in PAD rehabilitation and have been consistently reported in exercise intervention trials [8,14,18]. Randomised trials have shown that SET and structured home-based walking programmes can significantly improve walking performance and functional capacity in patients with PAD [12,13,45,46]. Additionally, combined strategies involving revascularisation and exercise therapy may provide complementary benefits in improving functional outcomes [12,47]. From a vascular perspective, clinically relevant outcomes such as patency, major adverse limb events (MALE), wound healing, and reintervention are critically important. However, these outcomes have been inconsistently reported across studies, limiting the ability to determine the impact of rehabilitation on vascular prognosis. Future studies should incorporate both functional and vascular outcomes to more comprehensively evaluate the benefits of postoperative rehabilitation.

Another important finding is that most rehabilitation programmes focus on walking-based exercises, with limited use of multimodal or comprehensive rehabilitation approaches. Although walking training remains the cornerstone of PAD rehabilitation, patients with advanced PAD or CLTI often present with skeletal muscle dysfunction, frailty, and reduced overall physical capacity [6,9]. Consequently, rehabilitation strategies incorporating resistance training, balance training, and functional mobility interventions may provide additional benefits. However, such multimodal approaches have been infrequently described in the included studies, highlighting an important gap in the current literature. This suggests that existing rehabilitation strategies may not fully address the complex functional impairments observed in this population.

A further question raised by this review is why exercise-based rehabilitation after lower-limb revascularisation remains poorly defined. Several factors may contribute. First, vascular and rehabilitation perspectives have traditionally emphasised different outcomes, with procedural success assessed mainly by vascular endpoints and rehabilitation studies focusing on functional recovery. Second, although major PAD guidelines recommend exercise following revascularisation, they provide limited guidance on postoperative-specific aspects such as initiation timing, exercise progression, and adaptation to wound status or disease severity. Practical implementation may also be complicated by wound care, mobility restrictions, and the transition from inpatient to outpatient or home-based rehabilitation. Finally, substantial heterogeneity in PAD severity, revascularisation procedures, frailty, comorbidities, and tissue loss makes standardisation challenging. Together, these factors may explain why postoperative exercise rehabilitation remains less standardised than exercise therapy for stable claudication.

Interpretation of the reported functional improvements requires particular caution in this setting. Most included studies did not report the technical outcome of the revascularisation itself—primary patency, restenosis, or reintervention during the rehabilitation period—so it cannot be determined whether an observed change in walking capacity reflects the exercise programme, the haemodynamic effect of a patent revascularisation, or the loss of that effect through restenosis. Spontaneous functional recovery following restoration of perfusion may account for a substantial proportion of the improvement observed, and in uncontrolled studies improvement cannot be attributed to the exercise intervention. In addition, unsupervised home-based programmes predominated among the included studies, yet few reported adherence in any quantitative form, so the exercise dose actually delivered is frequently unknown. The predominance of unsupervised programmes combined with sparse adherence reporting is itself a principal finding of this review and a major limitation of the current evidence base; concurrent reporting of vascular outcomes and adherence should be regarded as a minimum requirement for future studies.

Although CLTI is the population in which rehabilitation is most needed and least studied, the barriers in this group are substantial and require explicit consideration. Frailty and sarcopenia limit the tolerable exercise dose; tissue loss and wound infection restrict weight-bearing and ambulation and often dictate the rehabilitation schedule; markedly reduced exercise capacity renders conventional treadmill-based protocols and endpoints unsuitable; and the competing risks of major amputation and mortality complicate both the design and the interpretation of functional outcome studies. Rehabilitation strategies for CLTI therefore cannot simply be adapted from claudication protocols, and outcome measures other than maximal walking distance are likely to be required.

Comparisons drawn in this review between postoperative populations and patients with stable claudication are intended to provide context rather than to support inference. These populations differ substantially in disease severity, baseline functional capacity, wound healing status, comorbidity burden, and rehabilitation priorities, and evidence derived from stable PAD cohorts should not be assumed to transfer to patients undergoing revascularisation.

This study has some limitations. First, the included studies were heterogeneous in terms of study design, patient populations, and rehabilitation protocols, which limited direct comparisons. Second, as a scoping review, the aim was to map the existing literature rather than to quantitatively synthesise the effectiveness of rehabilitation interventions. Notably, most included studies focused on patients with claudication, while evidence specific to patients with CLTI remains limited. Furthermore, variability in outcome reporting across studies further restricts comparability and hinders evaluation of the impact of rehabilitation on vascular outcomes.

Two further characteristics of this literature qualify the map presented here. First, the included studies span more than three decades, and the applicability of the earliest studies to contemporary practice is limited by subsequent changes in revascularisation technique, perioperative care, and medical therapy; this temporal heterogeneity is an additional reason why the included evidence cannot be pooled. Second, the design limitations recorded in Table 3 indicate the prevailing methodological weaknesses of this literature: a control group or randomisation was absent in several studies, sample sizes were generally small, blinded outcome assessment was rarely feasible given the nature of the intervention, and adherence and vascular outcomes were seldom reported alongside functional outcomes.

Postoperative rehabilitation following lower-limb revascularisation remains inconsistently implemented despite its potential importance in functional recovery. The included studies indicate that early mobilisation and subsequent structured walking programmes have been implemented and appear feasible in selected postoperative populations. However, the available evidence does not establish the optimal timing of initiation, does not demonstrate safety with adequate statistical power given how rarely adverse events were reported, and does not permit comparison of effectiveness between timing strategies. The three-phase pattern described above should therefore be regarded as a preliminary, hypothesis-generating conceptual framework derived from 14 heterogeneous studies, intended to organise the existing literature and to guide the design of future research, rather than as a validated or recommended clinical pathway. Prospective validation is required before it could be used to inform clinical practice.

## 5. Conclusions

Exercise-based rehabilitation following lower-limb revascularisation in patients with PAD demonstrates substantial heterogeneity in programme characteristics, including exercise modality, timing of initiation, intensity prescription, supervision, and programme duration. Across the included studies, walking-based exercise was the most commonly implemented rehabilitation strategy, and walking capacity was the most frequently reported outcome measure. In contrast, clinically important outcomes—such as limb-related events, wound healing, and safety—have been reported less consistently. These findings highlight important gaps in the literature. Future research should focus on establishing standardised postoperative rehabilitation protocols and evaluating clinically meaningful outcomes beyond walking performance, particularly in patients with CLTI.

The current evidence map does not establish the effectiveness or safety of postoperative rehabilitation, the optimal timing of initiation, or the comparative effectiveness of different rehabilitation strategies. Future studies should use clearly defined postoperative populations, document revascularisation status and adherence, and report both functional and vascular outcomes together with intervention-related adverse events.

## Figures and Tables

**Table 1:** Characteristics of included studies evaluating exercise-based rehabilitation following lower-limb revascularisation in patients with peripheral arterial disease.

Study	Country	Design	Sample Size	PAD Severity	Revascularisation Procedure	Exercise Modality	Initiation Timing	Outcomes Reported	Intervention Category
Carlisi et al., 2017 [22]	Italy	Observational study	34 patients	CLTI or severe end-stage PAD	Infrainguinal lower-limb bypass surgery	Early postoperative mobility exercise and gait training	Mean 5.7 days postoperatively	Walking capacity, limb pain	(i) Early postoperative mobilisation
Jakubsevičienė et al., 2014 [23]	Lithuania	RCT	162 randomised; 117 completed 6-month follow-up (57 intervention, 60 control)	Fontaine II–III	Femoral–popliteal bypass grafting	Individualised supervised walking-based rehabilitation plus home-based non-supervised exercise therapy	Early postoperative period	6MWT, ABI, SF-36	(iii) SET + (ii) structured unsupervised
Kostewicz et al., 2019 [24]	Poland	RCT	30 patients	Fontaine IIb	EVT (balloon angioplasty ± stent)	Home-based walking exercise	Post-EVT	Walking capacity, QoL	(ii) Structured, unsupervised
Badger et al., 2007 [25]	United Kingdom	RCT	14 randomised patients	Short-distance IC or CLTI without tissue loss	Infrainguinal reconstructive surgery/Infrainguinal lower-limb bypass surgery	Supervised treadmill exercise	4 weeks postoperatively	Walking distance, ABI, QoL	(iii) SET
Bø et al., 2015 [26]	Norway	RCT	50 patients	Fontaine II IC	PTA ± selective stenting	Supervised aerobic interval training plus home-based exercise	Early post-PTA period	Walking capacity, limb haemodynamics, HRQoL	(iii) SET + (ii) structured unsupervised
Kruidenier et al., 2011 [27]	Netherlands	RCT	70 patients (35 intervention, 35 control)	Rutherford 1–4	EVT (balloon angioplasty ± stent)	Walking interval training/SET	Within 3 weeks post-EVT	Walking distance, ABI, QoL, patency	(iii) SET
Lundgren et al., 1989 [28]	Sweden	RCT	75 patients	IC	Peripheral vascular reconstruction	Supervised physical training	At randomisation; 6 weeks postoperatively in the combined arm	Symptom-free and maximal walking distance	(iii) SET
Kobayashi et al., 2023 [29]	Japan	Retrospective cohort	76 patients/93 limbs	Rutherford 2–3 IC	EVT (balloon angioplasty + stent)	Home-based walking exercise	Post-discharge	Patency, CD-TLR, MACE, survival	(ii) Structured, unsupervised
Kobayashi et al., 2021 [30]	Japan	Retrospective cohort	69 patients	Severe IC	Above-knee femoropopliteal bypass	Home-based aerobic exercise	Post-discharge	Patency, MACE, survival	(ii) Structured, unsupervised
Kato et al., 2022 [31]	Japan	RCT	44 patients	Rutherford 2–4	EVT with bare nitinol stenting	Monthly supervised treadmill walking plus home walking	Post-EVT outpatient follow-up	Restenosis, MALE, 6MWD, ABI	(iii) SET + (ii) structured unsupervised
Jakubsevičienė et al., 2019 [32]	Lithuania	Prospective controlled study	218 enrolled; 160 completed	Fontaine II–III	Femoral–popliteal bypass grafting	Individualised exercise programme	Early postoperative period	HRQoL (SF-36)	(iii) SET + (ii) structured unsupervised
Mazari et al., 2012 [33]	United Kingdom	RCT	178 patients	IC due to femoropopliteal disease	PTA	Supervised exercise programme	At randomisation (exercise-only arm) and 1 week post-PTA (combined arm)	Walking distance, QoL, restenosis, reintervention	(iii) SET
Koppe-Schmeißer et al., 2021 [34]	Germany	Prospective controlled study	41 patients	Rutherford 3	PTA	Home-based walking exercise	Post-PTA	Walking distance, ABI, oxidative stress markers	(ii) Structured, unsupervised
Mays et al., 2015 [35]	United States	RCT (exploratory pilot)	25 patients	Stable IC/post-EVT	EVT (prior endovascular therapy)	Community-based walking with training, monitoring, and coaching	Post-EVT/outpatient	Walking capacity (PWT), patient-reported outcomes	(iii) SET + (ii) structured unsupervised

Characteristics of studies evaluating exercise-based rehabilitation following lower-limb revascularisation in patients with peripheral arterial disease. Data include study design, population characteristics, revascularisation procedures, exercise interventions, timing of rehabilitation initiation, and reported outcomes. Abbreviations: ABI, ankle–brachial index; CD-TLR, clinically driven target lesion revascularisation; CLTI, chronic limb-threatening ischaemia; EVT, endovascular therapy; HRQoL, health-related quality of life; IC, intermittent claudication; MACE, major adverse cardiovascular events; MALE, major adverse limb events; PAD, peripheral arterial disease; PTA, percutaneous transluminal angioplasty; PWT, peak walking time; QoL, quality of life; RCT, randomised controlled trial; SET, supervised exercise therapy; SF-36, 36-Item Short Form Health Survey; 6MWD, 6 min walk distance; 6MWT, 6 min walk test.

**Table 2:** Characteristics of exercise-based rehabilitation interventions following lower-limb revascularisation in patients with peripheral arterial disease.

Study	Exercise Modality	Intensity Prescription	Session Duration	Frequency	Supervision	Programme Length	Setting
Carlisi et al., 2017 [22]	Passive joint mobilisation, active-assisted exercise, standing training, gait training	Progressive mobilisation according to patient tolerance	Approximately 30 min/session	Daily	Supervised physiotherapy by hospital physiotherapists	During hospital stay	Inpatient rehabilitation
Jakubsevičienė et al., 2014 [23]	Walking, treadmill training, stair climbing, lower-limb exercise	60–85% HRmax	Up to 45 min/session	6 days/week during rehabilitation → 3–5 days/week during home programme	Supervised rehabilitation-centre programme followed by home-based exercise	6 months	Rehabilitation centre → home programme
Kostewicz et al., 2019 [24]	Home-based walking exercise	60–120 steps/min to submaximal claudication pain	20–50 min/session	Three sessions/day, 3 days/week	Home-based self-managed programme with periodic physiotherapist monitoring	12 weeks	Home-based
Badger et al., 2007 [25]	Treadmill walking	Fixed-load treadmill protocol (2.0 miles/h, 10° incline)	Approximately 30 min/session	Two sessions/week	Supervised treadmill training by hospital clinical staff	6 weeks	Outpatient department
Bø et al., 2015 [26]	Aerobic interval training (walking, stair climbing, aerobic movements) plus home exercise	Borg scale 15–17 during high-intensity intervals	60 min/session	Two supervised sessions/week + one home session/week for 12 weeks, then three home sessions/week for 12 weeks	Supervised initial phase followed by home-based continuation	24 weeks total	Hospital-based + home-based
Kruidenier et al., 2011 [27]	Walking interval training	Submaximal claudication pain	Approximately 30 min/session initially	Two to three sessions/week plus daily home walking advice	SET by trained community physiotherapists	6-month follow-up	Community-based physiotherapy
Lundgren et al., 1989 [28]	Dynamic leg exercise (walking/cycle ergometer)	Exercise beyond claudication pain	Approximately 30 min/session	Approximately three sessions/week	Supervised physical training programme	At least 6 months	Outpatient rehabilitation
Kobayashi et al., 2023 [29]	Home-based aerobic walking exercise	Borg scale approximately 13	≥20 min/session	3–5 days/week	Unsupervised home-based exercise with outpatient follow-up	6 months of observation	Home-based with outpatient
Kobayashi et al., 2021 [30]	Home-based aerobic exercise	Borg scale approximately 13	≥20 min/session	≥3 days/week	Unsupervised exercise with counselling during outpatient visits	6 months (with longer-term adherence follow-up)	Home-based with outpatient
Kato et al., 2022 [31]	Treadmill walking combined with home walking	Near-maximal claudication pain; Borg 12–14 if no leg symptoms	30–45 min/session	≥3 sessions/week plus monthly supervised sessions	Monthly supervised session plus home-based training	12 months	Home-based with outpatient
Jakubsevičienė et al., 2019 [32]	Walking, treadmill training, stair climbing, lower-limb exercise	60–85% HRmax	Up to 45 min/session	6 days/week during rehabilitation → 3–5 days/week during home programme	Supervised rehabilitation followed by home exercise	6 months	Rehabilitation centre → home programme
Mazari et al., 2012 [33]	Closed-circuit training plus brisk walking	Progressive circuit training	NR	Three sessions/week	Supervised by physiotherapists or doctors	12 weeks	Hospital-based
Koppe-Schmeißer et al., 2021 [34]	Walking training	Near-maximal claudication pain	30–60 min/session	≥3–5 days/week	Unsupervised home-based walking programme	3 months	Outpatient
Mays et al., 2015 [35]	Community-based walking (training, monitoring, coaching)	Symptom-limited walking	Progressive walking sessions	Multiple sessions/week	Community-based with coaching/monitoring	14 weeks	Community-based

Summary of exercise-based rehabilitation programmes implemented in the included studies. Exercise characteristics included modality, intensity prescription, session duration, frequency, supervision, programme length, and rehabilitation setting. Abbreviations: Borg, Borg rating of perceived exertion scale; HRmax, maximal heart rate; NR, not reported; SET, supervised exercise therapy.

**Table 3:** Outcome measures reported across included studies evaluating exercise-based rehabilitation after lower-limb revascularisation in patients with peripheral arterial disease.

Study	Walking Capacity	Limb-Related Outcomes	Quality of Life	Adverse Events	Other Clinical Outcomes	Between-Group Comparison	Adherence Reported	Key Design Limitations
Carlisi et al., 2017 [22]	Ambulatory status	Limb pain improvement	NR	NR	Recovery course	No; single-arm design, within-group change only	NR; supervised in-hospital programme, no participation data	Single-arm observational; no control group
Jakubsevičienė et al., 2014 [23]	6MWD, PFWD	ABI	SF-36	Reoperation, above-knee amputation	NR	Yes; significant for 6MWD and PFWD (*p* < 0.001) and SF-36 physical (*p* < 0.001) and mental (*p* = 0.020) scores; ABI NS	NR	RCT; substantial attrition (162 randomised, 117 analysed)
Kostewicz et al., 2019 [24]	maximal claudication distance	NR	Study-specific QoL questionnaire	NR	NR	No; reported *p*-values refer to within-group change only	NR; self-completed walking diary only	RCT; small sample (*n* = 30)
Badger et al., 2007 [25]	PWT, MWD	Pre-/post-exercise ABI	SF-36, WIQ	Toe amputation, wound infection, graft occlusion	Lactate, haemodynamics	Yes; significant for MWD (*p* = 0.001), ABPI (*p* = 0.02) and WIQ (*p* = 0.013); SF-36 NS	Partial; 14 of 48 eligible patients participated (29%), none attended the 6-month follow-up	RCT; very small sample (*n* = 14)
Bø et al., 2015 [26]	6MWT, MWD, PFWD	ABI, pulse volume recording	SF-36, Claudication Scale	No major adverse events	NR	Yes; no significant between-group difference, both groups improved from baseline	Yes; 26 of 29 (90%) completed the programme and 21 of 26 (81%) attended >80% of sessions	RCT; small sample (*n* = 50)
Kruidenier et al., 2011 [27]	absolute claudication distance, functional claudication distance	ABI, restenosis, reintervention	SF-36, EuroQol 5-Dimension	Complications	WIQ	Yes; significant for ACD (mean difference 271.3 m, *p* = 0.011) and FCD (295.1 m, *p* = 0.003); WIQ and quality of life NS	Yes; mean of 20 sessions over 6 months, 7 patients discontinued early	RCT; open-label intervention
Lundgren et al., 1989 [28]	Symptom-free walking distance, MWD	ABI, toe pressure	NR	NR	Calf blood flow	Yes; significant for symptom-free and maximal walking distance (log-rank *p* = 0.0006 to 0.0001)	Partial; non-compliance described case by case, no attendance rate reported	RCT; dated revascularisation and medical management context
Kobayashi et al., 2023 [29]	Physical activity	CD-TLR	NR	MACE	Survival	Yes for vascular outcomes (3-year primary patency 97% vs. 71%, *p* = 0.002); functional outcomes not compared between groups	Yes; 52 ± 18 min/day, 5.8 ± 1.1 days/week; continuation 39% at 1 year and 33% at 3 years	Retrospective, non-randomised; continuation of exercise self-selected
Kobayashi et al., 2021 [30]	Physical activity (step count, International Physical Activity Questionnaire)	Primary/secondary patency	NR	MACE	Survival	Yes for vascular outcomes (5-year primary patency 97% vs. 61%, *p* = 0.0041); functional outcomes within-group only	Yes; 41 ± 22 min/day, 6.1 ± 1.1 days/week; continuation 41% at 1 year and 25% at 5 years	Retrospective, non-randomised; continuation of exercise self-selected
Kato et al., 2022 [31]	6MWD	Restenosis, CD-TLR, MALE	NR	Major bleeding, stent fracture	ABI	Yes; significant for 1-year freedom from in-stent restenosis (81.3% vs. 47.6%, *p* = 0.043); 6MWD and ABI NS	Partial; activity monitor provided to the intervention group only, control group not assessed	RCT; small sample (*n* = 44)
Jakubsevičienė et al., 2019 [32]	NR	NR	SF-36	NR	Risk factor-stratified HRQoL changes	Yes; significant for SF-36 domains (*p* < 0.001 to 0.035); walking capacity not assessed	NR	Partially randomised (control group self-selected); attrition (218 enrolled, 160 completed)
Mazari et al., 2012 [33]	initial claudication distance, MWD, patient-reported walking distance	ABI, restenosis, reintervention	SF-36, VascuQoL	NR	International Society for Cardiovascular Surgery outcome criteria	Yes; significant only for post-exercise ABPI (*p* = 0.002); claudication distances, MWD and quality of life NS	Partial; ≥85% attendance predefined as completion, actual attendance NR	RCT; open-label intervention
Koppe-Schmeißer et al., 2021 [34]	Initial claudication distance, Absolute claudication distance	ABI	NR	NR	Oxidative stress markers	Yes; no significant difference in initial (*p* = 0.839) or absolute (*p* = 0.421) claudication distance; ABI favoured the PTA group (*p* = 0.047)	NR; authors note the absence of compliance data	Non-randomised controlled; small sample (*n* = 41)
Mays et al., 2015 [35]	PWT, claudication onset time	NR	Patient-reported QoL (WIQ)	NR	NR	Yes; primary outcome PWT NS (*p* = 0.052); COT (*p* = 0.045) and WIQ (*p* = 0.001) significant	Yes; 81.9 ± 25.6% of prescribed sessions completed (344 of 420)	Pilot RCT; very small exploratory sample (*n* = 25)

Outcome measures reported in included studies evaluating exercise-based rehabilitation after lower-limb revascularisation in patients with peripheral arterial disease. Outcomes are grouped into walking capacity, limb-related outcomes, quality of life, adverse events, and other clinical outcomes. Abbreviations: ABI, ankle–brachial index; CD-TLR, clinically driven target lesion revascularisation; HRQoL, health-related quality of life; MACE, major adverse cardiovascular events; MALE, major adverse limb events; MWD, maximal walking distance; NR, not reported; PWT, peak walking time; QoL, quality of life; SF-36, 36-Item Short Form Health Survey; VascuQoL, Vascular Quality of Life Questionnaire; WIQ, Walking Impairment Questionnaire; 6MWD, 6 min walk distance; 6MWT, 6 min walk test.

## Data Availability

No new data were created or analyzed in this study. Data sharing is not applicable to this article.

## Supplementary Materials

The following supporting information can be downloaded at https://www.mdpi.com/article/10.3390/diseases14090311/s1: Table S1: Search strategy used for each database; Table S2: Severity-stratified summary of included studies.

## Abbreviations

The following abbreviations are used in this manuscript:

6MWD	6 min walk distance
6MWT	6 min walk test
ABI	ankle–brachial index
CD-TLR	clinically driven target lesion revascularisation
CENTRAL	Cochrane Central Register of Controlled Trials
CLTI	chronic limb-threatening ischaemia
EVT	endovascular therapy
HRmax	maximal heart rate
HRQoL	health-related quality of life
IC	intermittent claudication
MACE	major adverse cardiovascular events
MALE	major adverse limb events
MEDLINE	Medical Literature Analysis and Retrieval System Online
MWD	maximal walking distance
NR	not reported
PAD	peripheral arterial disease
PEDro	Physiotherapy Evidence Database
PFWD	pain-free walking distance
PRISMA-ScR	Preferred Reporting Items for Systematic Reviews and Meta-Analyses Extension for Scoping Reviews
PTA	percutaneous transluminal angioplasty
PWT	peak walking time
QoL	quality of life
RCT	randomised controlled trial
SET	supervised exercise therapy
SF-36	36-Item Short Form Health Survey
VascuQoL	Vascular Quality of Life Questionnaire
WIQ	Walking Impairment Questionnaire
