# Supplementary material for: Exercise-Based Rehabilitation Following Lower-Limb Revascularisation in Patients with Peripheral Arterial Disease: A Scoping Review
Source: Diseases. 2026 Aug 26;14(9):311. doi: 10.3390/diseases14090311 (PMC13605395; doi:10.3390/diseases14090311)
Supplement: Supplementary file 1 [file diseases-14-00311-s001.zip › diseases-4471761-supplementary.pdf]

(Supplementary File S1)

"Exercise-Based Rehabilitation Following Lower-Limb Revascularisation in Patients with Peripheral Arterial Disease: A Scoping Review"

Supplementary File – Figures

No supplementary figures.

Supplementary File – Tables

Supplementary Table S1: Search strategy used for each database

Supplementary Table S2: Severity-stratified summary of included studies

Supplementary Table S1. Search strategy used for each database

The search strategy was developed using a combination of controlled vocabulary terms and free-text keywords related to peripheral arterial disease, lower-limb revascularization, and exercise-based rehabilitation. The final searches were conducted 4 February 2026 across the included databases (PubMed: 4 February 2026; Embase: 4 February 2026; PEDro: 4 February 2026; CENTRAL: 4 February 2026).

Database	Search Strategy
**PubMed/MEDLINE**	**#1 Population**: ("Peripheral Arterial Disease"[Mesh] OR "Arterial Occlusive Diseases"[Mesh] OR "critical limb ischemia"[tiab] OR "chronic limb-threatening ischemia"[tiab] OR CLI[tiab] OR CLTI[tiab])<br>**#2 Revascularization**: ("Endovascular Procedures"[Mesh] OR Angioplasty[Mesh] OR "Stents"[Mesh] OR revascularization[tiab] OR revascularisation[tiab] OR "bypass surgery"[tiab] OR "vascular surgery"[tiab] OR postoperative[tiab] OR postsurgical[tiab])<br>**#3 Rehabilitation/Exercise**: ("Exercise Therapy"[Mesh] OR "Exercise"[Mesh] OR "Walking"[Mesh] OR "supervised exercise"[tiab] OR "exercise training"[tiab] OR "rehabilitation program"[tiab] OR "exercise program"[tiab] OR "walking training"[tiab] OR rehabilitation[tiab] OR training[tiab])<br>**#4 Final search**: #1 AND #2 AND #3
**Embase**	**#1 Population**: 'peripheral arterial disease'/exp OR 'arterial occlusive disease'/exp OR 'critical limb ischemia':ti,ab OR 'chronic limb threatening ischemia':ti,ab OR CLI:ti,ab OR CLTI:ti,ab<br>**#2 Revascularization**: 'endovascular surgery'/exp OR 'angioplasty'/exp OR 'stent'/exp OR revascularization:ti,ab OR revascularisation:ti,ab OR 'bypass surgery':ti,ab OR 'vascular surgery':ti,ab OR postoperative:ti,ab OR postsurgical:ti,ab<br>**#3 Rehabilitation/Exercise**: 'exercise therapy'/exp OR 'walking'/exp OR 'supervised exercise':ti,ab OR 'exercise training':ti,ab OR 'rehabilitation program':ti,ab OR 'exercise program':ti,ab OR 'walking training':ti,ab OR rehabilitation:ti,ab OR training:ti,ab<br>**#4 Final search**: #1 AND #2 AND #3

Database	Search Strategy
**PEDro**	Title and abstract search using the keywords: *peripheral arterial disease* AND *exercise training*. Filters applied: **Clinical trial**.
**CENTRAL (Cochrane Central Register of Controlled Trials)**	**#1 Population**: ("peripheral arterial disease" OR "arterial occlusive diseases" OR "critical limb ischemia" OR "chronic limb-threatening ischemia" OR CLTI)<br>**#2 Revascularization**: ("endovascular procedures" OR angioplasty OR "stents" OR revascularization OR revascularisation OR "bypass surgery" OR "vascular surgery" OR postoperative OR postsurgical)<br>**#3 Rehabilitation/Exercise**: ("exercise therapy" OR exercise OR "walking" OR "supervised exercise" OR "exercise training" OR "rehabilitation program" OR "exercise program" OR "walking training" OR rehabilitation OR training)<br>**#4 Final search**: #1 AND #2 AND #3

Supplementary Table S2. Severity-stratified summary of included studies

Severity Stratum	Studies	Typical Rehabilitation Focus	Common Intervention Features	Common Outcomes	Main Clinical Considerations
IC-dominant	Kostewicz et al., 2019; Bø et al., 2015; Lundgren et al., 1989; Kobayashi et al., 2023; Kobayashi et al., 2021; Mazari et al., 2012; Koppe-Schmeißer et al., 2021; Mays et al., 2015	Improve walking performance, physical activity, and quality of life	Walking-centred programmes; treadmill or home/community walking; symptom- or exertion-guided intensity; mainly outpatient or home-based	6MWD, treadmill walking distance, PWT, claudication onset time, ICD/ACD/PFWD/MWD, ABI, QoL/WIQ, patency/restenosis, reintervention	Exercise tolerance, cardiovascular risk management, restenosis surveillance, adherence
Mixed symptomatic PAD	Jakubsevičienė et al., 2014; Badger et al., 2007; Kruidenier et al., 2011; Kato et al., 2022; Jakubsevičienė et al., 2019	Functional recovery plus improvement in walking capacity and/or HRQoL	Multicomponent rehabilitation; walking with stair climbing or SET; earlier postoperative initiation; rehabilitation-centre or outpatient delivery	Walking capacity, ABI, HRQoL, patency/restenosis, reintervention	Individualised progression according to pain, wound/graft status, and postoperative recovery
CLTI-dominant	Carlisi et al., 2017	Restore basic mobility and protect the limb during early recovery	Early inpatient mobilisation; passive/assisted exercise, standing training, gait training; tolerance-based progression	Ambulatory status, limb pain, recovery of preoperative ambulatory status	Wound protection, frailty, infection risk, fall risk, amputation-related disability

Classification was based on PAD severity reported in the original studies. Studies spanning intermittent claudication and more advanced symptomatic PAD were grouped as mixed symptomatic PAD. Mazari et al. included PTA alone, supervised exercise alone, and combined PTA plus supervised exercise arms and

was classified as IC-dominant because all participants had intermittent claudication. The study by Mays et al. was added to the IC-dominant stratum based on the revised inclusion set.

Abbreviations: ABI, ankle–brachial index; ACD, absolute claudication distance; CENTRAL, Cochrane Central Register of Controlled Trials; CLI, critical limb ischaemia; CLTI, chronic limb-threatening ischaemia; HRQoL, health-related quality of life; IC, intermittent claudication; ICD, initial claudication distance; MEDLINE, Medical Literature Analysis and Retrieval System Online; MWD, maximal walking distance; PAD, peripheral arterial disease; PEDro, Physiotherapy Evidence Database; PFWD, pain-free walking distance; PTA, percutaneous transluminal angioplasty; PWT, peak walking time; QoL, quality of life; SET, supervised exercise therapy; WIQ, Walking Impairment Questionnaire; 6MWD, 6-min walk distance.
